# Proteasome stress triggers differential cellular responses of neural cells

**DOI:** 10.1186/2193-1801-4-S1-P40

**Published:** 2015-06-12

**Authors:** Peter Racay, Ivana Pilchova, Katarina Klacanova, Dusan Dobrota

**Affiliations:** Jessenius Faculty of Medicine, Martin, Slovak Republic

**Keywords:** ischemia, cell death, proteasome

Ubiquitin-proteasome system (UPS) represents important intracellular system controlling protein quality and intracellular signalling. Overload or dysfunction of UPS leads to proteasome stress that is implicated in mechanisms of neurodegeneration associated with neurodegenerative diseases, e. g. Parkinson and Alzheimer disease. Proteasome stress is also considered as the main cause of delayed neuronal death observed after transient global brain ischemia. Despite significant progress made to date, the exact mechanism and selectivity of cell death induced by proteasome stress after global brain ischemia is still not completely understood. The aim of our work was to study effect of proteasome stress on cell viability, stress response as well as on mechanism of death of neuroblastoma SH-SY5Y and glioblastoma T98G cells. Proteasome stress was induced by treatment of cells with bortezomib, inhibitor of proteasome 26S complex. Neuroblastoma cells were more sensitive to bortezomib than glioblastoma cells and death of neuroblastoma cells occurred significantly faster than death of glioblastoma cells. With respect to cellular response, treatment of both SH-SY5Y and T98G cells with bortezomib was associated with accumulation of polyubiquitinylated protein aggregates and increased expression of HSP70. With respect to cell death mechanism, we have documented bortezomib-induced release of cytochrome c from mitochondria and activation of caspase 3 in SH-SY5Y cells. In T98G cells, bortezomib induced activation of caspase 4 but not caspase 3 and did not induce release of cytochrome c from mitochondria. Our results indicate that proteasome stress affects neural cells in different way but does not answer the question about selectivity and delay of cell after global brain ischemia.

